# Mesoscale atmosphere ocean coupling enhances the transfer of wind energy into the ocean

**DOI:** 10.1038/ncomms11867

**Published:** 2016-06-13

**Authors:** D. Byrne, M. Münnich, I. Frenger, N. Gruber

**Affiliations:** 1 Environmental Physics, Institute of Biogeochemistry and Pollutant Dynamics, ETH Zurich, CHN E 23.2, Universitatstrasse 16, Zürich 8092, Switzerland; 2 Center for Climate Systems Modeling (C2SM), ETH Zurich, Zürich 8092, Switzerland; 3 Princeton University, Princeton, New Jersey 08544, USA; 4 GEOMAR, Helmholtz Centre for Ocean Research, Kiel 24105, Germany

## Abstract

Although it is well established that the large-scale wind drives much of the world's ocean circulation, the contribution of the wind energy input at mesoscales (10–200 km) remains poorly known. Here we use regional simulations with a coupled high-resolution atmosphere–ocean model of the South Atlantic, to show that mesoscale ocean features and, in particular, eddies can be energized by their thermodynamic interactions with the atmosphere. Owing to their sea-surface temperature anomalies affecting the wind field above them, the oceanic eddies in the presence of a large-scale wind gradient provide a mesoscale conduit for the transfer of energy into the ocean. Our simulations show that this pathway is responsible for up to 10% of the kinetic energy of the oceanic mesoscale eddy field in the South Atlantic. The conditions for this pathway to inject energy directly into the mesoscale prevail over much of the Southern Ocean north of the Polar Front.

## Abstract (web summary)

The precise mechanism for wind energy input into the ocean at mesoscales remains uncertain. Here, using a high-resolution atmosphere-ocean model of the South Atlantic, the authors show that a mesoscale conduit associated with oceanic eddies is responsible for up to 10% of kinetic energy transfer.

## Graphical abstract

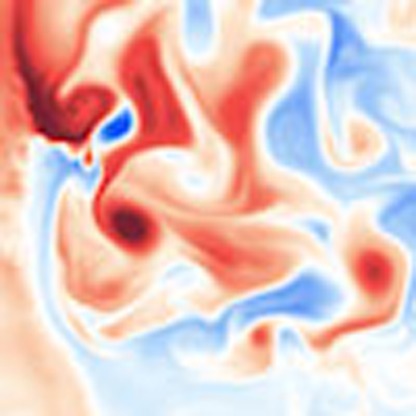

It has become increasingly apparent that oceanic mesoscale processes are a crucial element of the large-scale oceanic circulation with major implications for climate and ocean biogeochemistry^1^,^2^,^3^,^4^,^5^. This is especially the case for the Southern Ocean, where eddies and other mesoscale processes such as meanders and filaments partially compensate the wind-driven circulation via an eddy-induced overturning circulation, whose balance determine the net Meridional Overturning Circulation^6^,^7^,^8^. This compensation is dependent on the kinetic energy (KE) of the mesoscale eddy field^7^. Thus, changes to this eddy KE potentially have substantial implications for the uptake of carbon^9^,^10^,^11^,^12^ and heat^13^,^14^ in the Southern Ocean. One strategy to include mesoscale processes in weather and climate models is to resolve them directly by increasing the resolution of the ocean component. This captures the main energy pathway into the ocean mesoscale eddy field, that is, the large-scale potential energy generation by wind and buoyancy, followed by baroclinic instability. However, in such an approach, any energy flux into the ocean emerging from the mesoscale interaction between the ocean and atmosphere remains unresolved.

A well-studied mesoscale energy pathway involves the mechanical coupling between oceanic mesoscale structures and the overlying atmosphere^15^,^16^,^17^,^18^. This pathway emerges, as the wind stress exerted by the atmosphere on the ocean depends not only on the motion of the air but also on the relative motion of the surface ocean^15^. Relative to a situation where this mechanical coupling is not included, the momentum flux into the ocean is reduced whenever the ocean current and the wind are aligned. Correspondingly, the flux is enhanced when the wind and the ocean currents oppose each other. Owing to the quadratic dependence of the wind stress on the wind speed, the net effect is a reduction of the energy flux into the ocean, an effect that has been termed ‘mechanical damping'^15^,^16^,^17^,^19^. Regional model simulations suggest that this reduction may be as large as 17–27%, depending on the region^16^,^18^.

Here we demonstrate that this mechanical damping can be outweighed by a second mesoscale energy pathway that involves the thermodynamic coupling between oceanic mesoscale features and the overlying atmosphere^20^,^21^,^22^,^23^,^24^,^25^,^26^. This thermodynamic pathway, which has received comparatively little attention so far^2^,^27^, is driven by the sea-surface temperature (SST) anomalies associated with the oceanic mesoscale field. Through their anomalous heat fluxes, positive SST anomalies destabilize the atmosphere above them, causing more momentum from higher up in the atmospheric boundary layer to be mixed downward, leading to stronger surface winds^22^ (Fig. 1). Cold SST anomalies have the opposite effect, stabilizing the overlying atmosphere and reducing the near-surface winds. Our results indicate that this thermodynamic coupling provides an energy conduit that modifies the oceanic mesoscale energy field substantially. Provided a wind gradient of the right sign, this thermodynamic pathway can fully compensate for the mechanical damping effect and may actually contribute up to 10% of the KE in the mesoscale field.

## Results

### Mesoscale air–sea coupling hypothesis

One potential reason for the lack of consideration of this thermodynamic pathway in the past is the common assumption that at the scale of an ocean mesoscale feature, the background wind is constant. Under this prevailing view, the ocean mesoscale-induced thermodynamic response of the atmosphere results in only little net change in the energy transfer. In the case of a perfectly symmetric eddy, that is, a coherent mesoscale vortex, the net flux is actually zero, as the increased work on one side of the eddy is exactly balanced by the reduced work on the other side. However, ocean eddies rarely live in an area of constant background wind, in particular not in the Southern Ocean^28^ (Fig. 2a).

**Figure 1:**
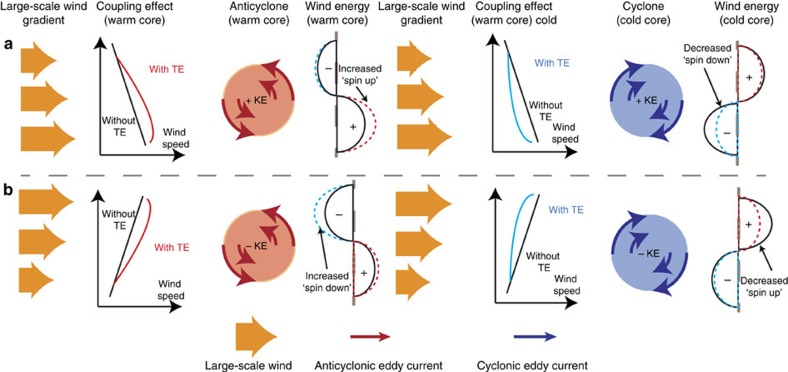
Proposed modification of the exchange of wind energy between the atmosphere and ocean. (**a**) Cases for a negative wind gradient (increasing from north to south). (**b**) Cases for a positive wind gradient. TE, thermodynamic effect. (**a**) Response for a warm-core anticyclonic eddy and for a cold-core cyclonic eddy separately (both in the southern hemisphere). The eddy-associated anomalies in SST modify the overlying winds and hence the wind stress exerted onto the ocean (coupling effect). In the case of a warm-core eddy lying within a negative wind gradient (**a**), this enhances the energy transfer induced by the mechanical coupling between the eddy current and the wind on the south side of the eddy, whereas it affects the mechanical spin down on the northern side little (wind energy). The net effect is thus an increase in the net energy transfer into the ocean. The other cases operate analogously, resulting in regions with a negative wind gradient tending to energize the oceanic eddies (**a**), whereas regions with a positive gradient having the opposite effect (**b**).

The potential impact of the consideration of a lateral wind gradient over the scale of an ocean eddy (compared with the no gradient case; see, for example, Fig. 1 in ref. 17) is conceptually illustrated in Fig. 1. A negative wind gradient (defined as an increase in wind speed from north to south) results in either a ‘spin-up' (anticyclonic) or ‘spin down' (cyclonic) forcing that is active even without taking into account the feedback of the SST anomalies on the atmosphere. If we now add the change in the winds caused by the anomalous SSTs associated with the underlying eddies, this effect gets altered further. The net result is an increase in KE for both warm and cold core eddies that reside in a negative wind gradient and a decrease in KE when they are located in a positive wind gradient.

The wind gradient over the scale of an ocean eddy also has an effect on the mechanical damping, as this effect will now be stronger on the side of the eddy with the stronger wind. However, we propose that as long as the background wind across the eddy changes more than the ocean surface current ∼*O* (0.5 m s^−1^), the thermodynamic effect will dominate the energy transfer at these scales.

### Modelling the mesoscale air–sea coupling

To test our hypothesized thermodynamic pathway associated with a background wind speed gradient, we employed a high-resolution (10 km) coupled atmosphere–ocean regional model (COSMO^29^–Regional Oceanic Modeling System (ROMS)^30^)^22^ in the South Atlantic, a region with high eddy activity. We undertook three numerical model experiments, to quantify the thermodynamic effect and to contrast it with the mechanical dampening one. In the first simulation (mesoscale mechanically coupled, MMC), we only consider the mechanical damping effect, that is, the ocean surface velocity is sent to the atmospheric model every time step for the calculation of the wind stress, but the atmosphere is not aware of any eddy-scale SST anomalies. In the standard simulation (mesoscale fully coupled, MFC), we additionally allow for the thermodynamic effect, that is, the wind changes in response to the underlying SST anomalies. Here, the ocean model provides at each time step the SST and ocean surface velocity to the atmosphere, and the atmosphere returns the net heat, freshwater and momentum fluxes. To isolate the role of the mechanical energy pathway, we conducted a third simulation (mesoscale thermodynamically coupled, MTC), where only the thermodynamic response of the atmosphere is allowed, that is, the ocean surface current is omitted from the wind stress calculation. All simulations produce a realistic distribution of eddies, with several hundred warm- and cold-core eddies of varying sizes (20–300 km) populating the South Atlantic domain (Fig. 1b) at any given time, in excellent agreement with observations^22^.

**Figure 2:**
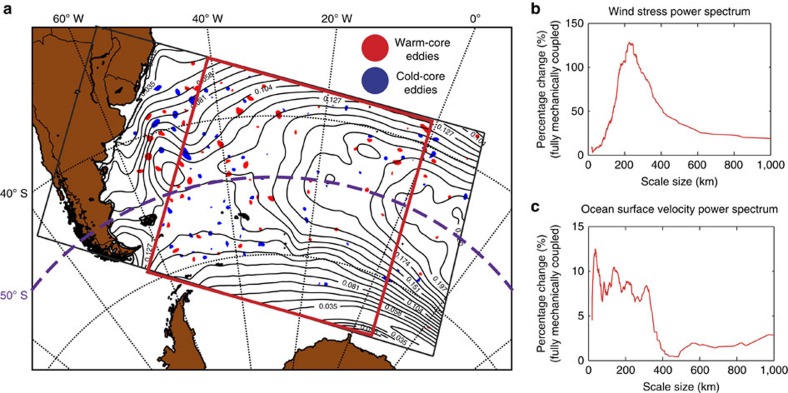
Model domain wind stress and ocean KE power spectrum. (**a**) Map of the model domain, showing a snapshot of the detected warm (red) and cold (blue) core eddies. The contours depict the austral winter climatological wind stress magnitude taken from ERA interim. The red square indicates the region used for the spectral analysis. The dashed line at 50°S separates the northern region characterized by a negative wind gradient from the southern region that has a positive wind gradient. (**b**) Relative change of the wind stress power spectrum between the fully coupled and the mechanically coupled simulation (MFC–MMC) computed over the red square.(**c**) As in **b**, but for the relative change of the ocean KE power spectrum.

The simulations reveal that the thermodynamic effect, which we isolate by contrasting the fully coupled with the mechanically-only coupled simulation (MFC–MMC), results in a substantial change of the oceanic mesoscale energy field. This is illustrated in Fig. 2c by the difference of the ocean KE power spectra from these two simulations, calculated over the entire western South Atlantic (the region defined by the red box (Fig. 2a)). This difference reveals that the fully coupled run contains ∼10% more KE in the mesoscale range (20–400 km) compared with the mechanically coupled run. In addition, the change in wind forcing between the two simulations, as shown from the time-averaged, power spectrum of the wind stress (Fig. 2b), reveals that changes to the wind forcing are mostly restricted to spatial scales of ∼20–600 km. This indicates that the ocean KE change is driven by changes in the mesoscale wind forcing and not by changes in the transfer of energy from larger scales.

In contrast, our simulations reveal only a modest dampening effect from the mechanical energy pathway, isolated by contrasting the fully coupled with the thermodynamically-only coupled simulation (MFC–MTC). In this case, we find a 3% reduction in the energy contained in the oceanic mesoscale field. Thus, in our South Atlantic domain, the thermodynamic pathway clearly outweighs the mechanical one.

### Thermodynamic coupling at the ocean eddy scale

To understand the mechanisms behind the thermodynamic coupling effect energizing the oceanic mesoscale, we first focus on the contribution of eddies. This choice is justified by eddies being highly abundant in our analysis region^28^ and also by our hypothesis having been developed for such coherent vortices. We will demonstrate that eddies largely explain the effect, suggesting that the contribution of the other mesoscale features, such as filaments, are of lesser importance.

Our conceptual model for how the thermodynamic coupling at the mesoscale has an impact on the net energy transfer from the atmosphere to the ocean (Fig. 1) suggests that the overall gain of eddy KE in our South Atlantic domain must come from a prevalence of spin-up conditions over spin-down conditions. The climatology of the wind stress magnitude in Fig. 2a (black contours) reveals that both conditions occur in our domain, that is, a region of negative meridional wind gradient north of ∼50°S, representing a ‘spin-up' region, and a region of positive wind gradient south of ∼50°S, forming a ‘spin down' region. Thus, the domain-wide increase indicates that the energy gain in the ‘spin-up' region exceeds the energy loss in the ‘spin-down' region, probably due to more eddies populating the North.

To test this explanation and our hypothesis, we split the domain into the two distinct regions: north and south of 50°S, that is, the ‘spin-up' and ‘spin-down' regions (purple dashed line Fig. 2a). In each of these two domains, we disentangle the ocean eddy response by using a semi-Lagrangian frame of reference. Mean composites of the wind energy input,and KE are computed from a rectangle centred on each eddy we identified in the model using two methods^21^,^22^. Each eddy is scaled to a common size and rotated so that the background wind falls along the *x* direction^21^.

**Figure 3:**
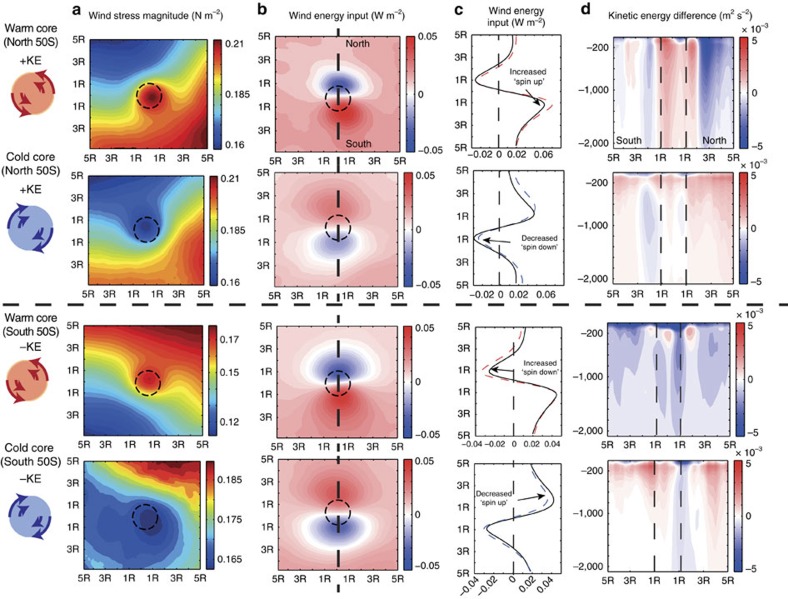
Model results of the modification of the wind energy transfer at the mesoscale. Composite eddy maps, separated by wind stress gradient (north and south of 50°S) and cold- and warm-core eddies. (**a**) Eddy-induced wind stress change above the eddies from the fully coupled simulation (MFC). (**b**) Wind energy input for the fully coupled simulation showing the dipole structure resulting from the coupling between the ocean and atmosphere; (**c**) wind energy input along the chord (vertical line in **b**) separately for the fully coupled (MFC dashed red) and the mechanically coupled simulation (MMC solid black). (**d**) Vertical section of the difference in ocean KE between the fully and mechanically coupled simulation (MFC–MMC) (along the same vertical line in **b**), showing an increase for both warm- and cold-core eddies in the northern and a decrease in the southern wind gradient region.

The wind stress composites associated with warm- and cold-core eddies (Fig. 3a) clearly show the positive and negative wind stress gradients across eddies for both the ‘spin-up' and ‘spin-down' regions. The dipole structure of the mean energy input reflects the respective rotation directions of the eddies and their interaction with the background westerlies (Fig. 3b).

**Figure 4:**
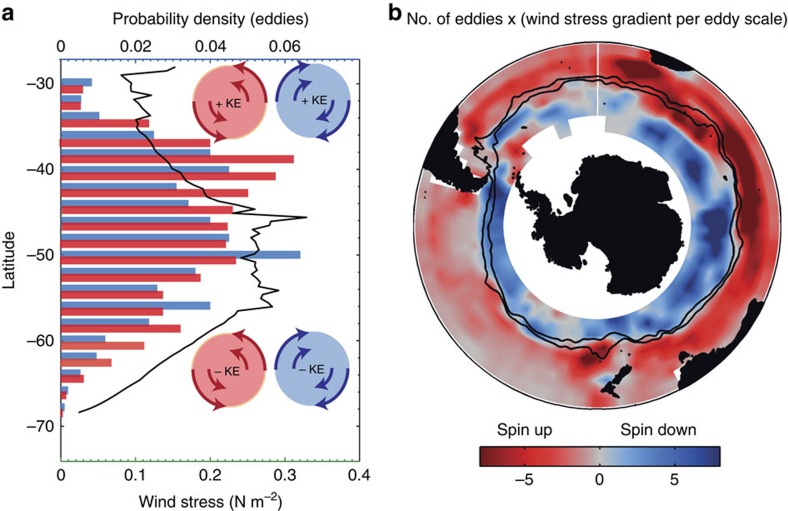
Satellite observations and regions for the mesoscale thermodynamic pathway. (**a**) Normalized probability distribution of warm (red)- and cold (blue)-core eddies as a function of latitude compared with the mean, time-integrated wind stress (black solid line). (**b**) Southern Ocean map of the product of the average wind stress gradient across an eddy within each 4° (longitude) times 2° (latitude) grid box and the number of eddies within this box. The wind stress was computed from SeaWinds on QuikSCAT and the eddies were identified from satellite sea surface height anomaly data (AVISO) over the period June 2002 to November 2009 (see Methods); shown are only values that are significantly different from 0 at the 1% confidence level (as judged by a *t*-test). The mean positions of the Subantarctic and Polar Fronts are shown as solid black lines; reddish colours show anticyclonic (negative gradient) wind shear situations, bluish colours show cyclonic (positive gradient) wind shear situations; the former represent ‘spin-up' conditions with respect to the coupling effect discussed here and the latter represent ‘spin-down' conditions.

In light of our proposed mechanism, we expect the difference in energy input to follow the strength of the background wind^22^ (Fig. 1). Figure 3c shows this difference along the chord (south to north) indicated by the dashed line in Fig. 3b. For the negative wind gradient north of 50°S, there is an increase of the energy input for warm-core (spin-up) and a decrease of energy loss (spin down) for the cold-core eddies (dashed relative to solid line in Fig. 3c). This leads to an intensification for both eddy types north of 50°S, that is, an increase in their KE, as predicted (Fig. 3d). The vertical sections in Fig. 3d additionally reveal that this enhancement is surface intensified, supporting the hypothesis of a direct scale-to-scale forcing from the overlying wind. South of 50°S, the positive wind gradient produces the opposite effect, increasing the ‘spin down' of warm-core and decreasing the ‘spin-up' of cold-core eddies, resulting in a reduction in the KE (Fig. 3c,d). We note the weaker signal for the cold core eddy in the second row of Fig. 3d. This is mainly due to a peak in the cold-core eddy abundance residing in no wind gradient (Fig. 4a), resulting in a weaker signal compared with the background (also seen in the wind energy input difference). A stronger signal is recovered if one only chooses eddies that reside in the positive wind gradient (Supplementary Fig. 1).

We tested the robustness of these composites by subsampling from all identified eddies a set of every odd or even number, resulting in only marginal changes in the curves (Supplementary Fig. 2a,b). The results are also independent of the eddy detection method^22^.

### Mesoscale energy budget

To demonstrate quantitatively that the diagnosed increase/decrease in the KE at the eddy scale stems from the thermodynamic energy pathway, we consider the local energy budget difference between the fully coupled and mechanically coupled simulations. Given our previous finding that the change in eddy KE stems directly from the energy input at the mesoscale (see Fig. 2b,c), we interpret that the difference of KE we detect in the ocean eddy composite (Fig. 3d) is a direct response to the difference in the wind energy input (Fig. 3c). We furthermore assume that changes to potential energy, bottom friction and energy transport would not play a major role. This assumption is justified given that all simulations started from the same initial conditions and ran over a period of 3 months only, that is, long enough to fully establish the mesoscale atmosphere–ocean interactions of interest here, but short enough for the other contributions to remain largely unchanged. Thus, we would expect that the difference in the local KE at the eddy scale between the two simulations should be largely explained by change in the wind forcing only, that is,


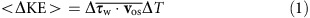


where ΔKE is the KE difference between the fully coupled and mechanically coupled simulation (MFC–MMC). The expression **τ**_w_.**v**_os_ is the wind power input computed from the dot product of the wind stress **τ**_w_ and the ocean surface velocity **v**_os_ and Δ*T* represents the average lifetime of an eddy. Angled brackets and over bar denote volume and surface integrals, respectively.

We estimate 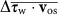 from the difference in the wind energy input composite and integrate over an area *πR*^2^ in eddy-normalized coordinates. *R* is the average eddy size chosen here as *R*=35 km for the region north of 50°S and *R*=25 km south of 50°S. For the eddy lifetime, we take a range of 30–60 days. To compute <ΔKE>, we take the energy difference from the eddy composites and integrate it over the volume of one eddy, that is, *πR*^2^*h*, where *h* is a depth of 2,000 m.

With these inputs and assumptions, we find a good closure of the mesoscale energy flux budget over the average eddy in both the spin-up and spin-down regions. In the case of warm-core eddies, the energy input difference, 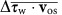, amounts to +4.2 MW in the spin-up region and −2.1 MW in the spin-down region. These values compare very well with the average change in the KE of the average eddy over this time period, that is, 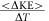 of +2.9 MW (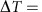
60 days) to +5.9 MW (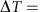
30 days), respectively, for the spin-up region and a value 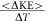
of −1.4 to −3.0 MW for the spin-down region. The absolute numbers depend highly on our estimates of the mean eddy parameters, but the large number of analysed eddies (∼20,000) and the low sensitivity to subsampling gives us some confidence in our results. Bearing in mind the uncertainties, the fact that we find a good closure confirms in both regions our hypothesis that the eddy-induced anomalous thermodynamic energy flux can account for the majority of the change in the KE in the oceanic mesoscale.

### Dominance of the thermodynamic pathway

Given the dominant contribution of eddies to the thermodynamic energy pathway, the South Atlantic domain-wide ∼5–10% increase in KE must stem then largely from differences in the eddy abundances in the two regions. Indeed, there are substantially more warm- and cold-core eddies in the northern spin-up region, compared with the southern spin-down one (Fig. 4a). As a result, the thermodynamic pathway has a clear signal in the ‘spin-up' region and increases the KE by ∼+12%. However, in the ‘spin-down' region, the signal is less clear and although the spin-down effect is detectable at the eddy scale in this region, there is no significant signal for the thermodynamic pathway for the integrated KE in the ‘spin-down' region (solid green line, Supplementary Fig. 3).

There are several reasons for the reduced effect in the high-latitude, South Atlantic spin-down region. First, the eddies are smaller in size compared with eddies in the northern region. Second, during this period ice cover starts to encroach on this region and hinders the extraction of energy directly from the mesoscales via this mechanism. Third, owing to the simple partitioning of north and south of 50°S, there is still a portion of the domain that remains ‘spin up' south of 50°S compensating for the ‘spin down' (see Fig. 4b).

In contrast to the thermodynamic pathway, the mechanical energy pathway reduces the mesoscale energy content by ∼3% in both regions, that is, it is strongly outweighed by the thermodynamic pathway in the spin-up region (dashed purple line, Supplementary Fig. 3). Additional support for the dominance of the thermodynamic pathway over the mechanical pathway in the South Atlantic comes from the analysis of the wind stress curl pattern above eddies and their comparison with satellite observations. The mechanical pathway leads to a monopole curl pattern, as this response is determined largely by the ocean surface current only. This monopole signal is dwarfed by the dipole structure that results from the additional consideration of the thermodynamic effect in the fully coupled simulation. In addition, it is exactly these dipole structures that are found by a corresponding analysis of the wind stress curl pattern over eddies from satellite observations in the South Atlantic and the entire Southern Ocean^22^,^31^ (Supplementary Fig. 4a,b).

Finally, we compare the magnitude of this new energy pathway with the standard baroclinic instability route by analysing the coherence between wind and surface currents (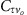, see Methods). Assuming a linear response between the wind and ocean velocity, the coherence spectrum estimates the scale-to-scale power transfer. Supplementary Fig. 5 shows that with consideration of the thermodynamic energy pathway (red line), the energy contained at scales 50–150 km that may be explained via wind doubles compared with a case with the mechanical damping only (black line). The figure also reveals a strong scale dependence of the thermodynamic pathway. Although this pathway explains only ∼2% of the energy at the 50–150 km scale, it is responsible for up to 8% at scales larger than ∼100 km (the remaining percentages are assumed to have been supplied via the standard energy pathway, that is, downward cascade from the larger scales).

The fact that our new thermodynamic mechanism is scale dependent is consistent with our proposed hypothesis. Indeed, subsampling only eddies >75 km in diameter when computing the composites in Fig. 3 results in very little change to the result, indicating that the majority of the signal is coming from larger eddies. Further, by binning the magnitude of the asymmetry in wind energy input across eddies as a function of the size of the eddy, we see a clear scale dependence as well. In this case, larger eddies have an increased asymmetry in their wind forcing (not shown).

So far, by focusing exclusively on eddies, we have neglected the possible contribution of other ocean mesoscale structures in the 20–400 km scale range such as meanders, filaments and jet-like structures. Our analyses suggest that their contribution is small, as our proposed mechanism acting on eddies offers a plausible explanation for the majority of the differences in KE. The eddies account for the KE difference between the spin-up/down regions of the domain and they also explain the integrated total change in energy across the domain. Thus, we have good reasons to believe that our eddy-focused mechanism is the dominant effect. We leave it to future studies to determine the specific role played by the other mesoscale phenomena.

### Regions for the mesoscale thermodynamic pathway

The thermodynamic energy pathway is important in any region where the abundance of eddies is high and where these eddies are embedded in a strong lateral gradient in the wind stress. Both conditions are met over much of the Southern Ocean. To quantify this effect more precisely, we analysed >600,000 eddies (with lifespans of 2 weeks or more) identified from satellite sea-surface height anomaly data (AVISO) over the period June 2002 to November 2009 (see Methods). Approximately 30% of the eddies (∼200,000) exist in a strong gradient with an absolute value exceeding 0.01 N m^−2^ per eddy diameter, similar to the average model eddy in Fig. 3a, that is, they experience across their area a change of wind stress of roughly 10% and thus provide an excellent conduit for the mesoscale exchange of energy between the atmosphere and the ocean (see Supplementary Fig. 6a–c). The spatial extent is illustrated in Fig. 4b, which shows the product of the number of eddies instances per 4° (longitude) times 2° (latitude) bin with the average lateral wind gradient over these eddies. This reveals that spin-up conditions (red areas) prevail over large regions of the Southern Ocean north of the Polar Front, and that the energy transfer is likely to be the highest in the Indian Ocean sector given the high number of eddies there^28^. In contrast, spin-down conditions are mostly located south of the Polar Front and, if we extrapolate our results from the South Atlantic to the other sectors, we similarly expect that the integrated reduced energy flux in these regions is much smaller than the enhanced energy flux in the spin-up regions to the north. Thus, it is clear that this mechanism will be important for large regions of the Southern Ocean and most probably anywhere where a strong gradient in the background wind field exists.

## Discussion

So far, direct atmosphere–ocean interactions at the mesoscale were generally believed to play little role and, if anything, lead to a reduction of the energy transfer from the atmosphere to the ocean through mechanical damping^15^,^16^,^17^,^19^. Here we propose a revision to this view, as we have demonstrated that in the Southern Ocean, owing to the presence of large wind gradients, our newly uncovered thermodynamic pathway is outweighing the negative effect of the mechanical damping and may actually result in a net powering of the mesoscale field.

The thermodynamic pathway we described is likely to get stronger in the future with the projected increases and shifts in Southern Ocean wind^32^,^33^,^34^. ‘Spin-up' and ‘spin down' zones will intensify with increased winds, thus further energizing the oceanic mesoscale in the regions north of the Antarctic Circumpolar Current, whereas dampening the mesoscale activity in the Antarctic Zone south of the Polar Front. Furthermore, more eddies may populate the ‘spin-up' side of the wind stress gradient with the projected poleward shift of the wind maxima by the end of the century^34^.

To quantify this new pathway we have investigated the transient response of the ocean. However, the question remains as to how this mesoscale energy input at the ocean surface affects the large-scale ocean circulation, which is difficult to predict given the many nonlinear interactions that transfer and dissipate energy among different scales. However, these changes will probably modify vertical mixing and potentially the degree to which the eddy-induced counter flow compensates the increased northward Ekman flow^7^,^11^, which greatly affects the oceanic uptake of CO_2_ and heat from the atmosphere^10^,^11^,^12^,^13^,^14^.

## Methods

### Coupled regional atmosphere–ocean model

We employed a coupled regional atmosphere–ocean model^22^, COSMO^29^, for the atmospheric component and the ROMS^30^ for simulating the ocean. Both the atmosphere and the ocean models used the same horizontal grid with rotated coordinates in reference to a north pole geographic location of 37N, 10W. The model domain spans ∼7,000 km by 3,500 km and has a resolution of (0.09° × 0.09°)≈10 km^2^. The atmosphere was configured with 40 model layers in the vertical, whereas the ocean model has 42 vertical levels.

The atmospheric model component was initialized with ERA-Interim^35^ reanalysis data for 1 June 2004. The ocean part was initialized with climatological January temperature and salinity based on SODA^36^ and spun up from rest for 2 years with 6-hourly surface flux forcing (wind stress, net heat and fresh water) from ERA-Interim 2004 reanalysis^35^ (2004 forcing repeats during spin-up). Ocean lateral boundary conditions were provided from 30 year mean climatological fields from SODA. After spin-up, the atmosphere and ocean models were coupled. Each of the three 3-month simulations started on 1 June.

Synchronized exchange of data fields between the component models was handled via the OASIS3 coupler^37^. Exchanged fields were sent at every time step (60 s) with no grid or time interpolation between model components. The exchanged fields for each simulation are described below.

MMC, ocean surface velocity was sent to the atmospheric model every time step for calculation of the wind stress. Monthly mean, SST from ERA-Interim was used by the atmospheric model for the calculation of net heat, freshwater and momentum fluxes at each time step. This results in the thermodynamic energy pathway being excluded.

MFC, standard fully coupled setup, with the ocean model sending SST and ocean surface velocity, and the atmosphere returning net heat, freshwater and momentum fluxes at each time step.

MTC, as in MFC, but the ocean surface velocity is not included in the calculation of the wind stress returned by the atmospheric component model, that is, the mechanical energy pathway is excluded.

We employed an updated version of the COARE (Coupled Ocean–Atmosphere Response Experiment)^38^ algorithm for the calculation of the turbulent fluxes of heat, moisture and momentum between the atmosphere and ocean, where ocean SST and surface velocity fields are used for the wind stress calculations.

Vertical mixing of momentum and scalars in the ocean was parameterized via a KPP scheme^39^.

### Eddy identification

We employed two automated eddy detection methods: the first is based on the Okubo–Weiss parameter^40^,^41^, where eddies are identified from sea-level height anomalies, and the second is a vector geometry based eddy detection method where eddies are identified based on their velocity sign reversal^42^.

To create the eddy composites for the observed atmospheric and oceanic variable, we used the results from the Okubo–Weiss detection scheme and computed the fields by averaging across all detected eddies after scaling and rotating them^21^. The observed wind speed data stem from collocated satellite data provided by SeaWinds on QuikSCAT. Ocean velocities were estimated from geostrophy for wind energy input composites.

Model-based atmospheric and oceanic variable composites were computed from 6-hourly model output fields using both Okubo–Weiss and vector-geometry eddy detection algorithms for comparison.

### One-dimensional wavenumber spectra calculation

The power spectrum displayed in Fig. 1b,c were calculated as follows (here described for the wind stress; however, the same calculation is performed for the ocean with wind stress,**τ**, replaced by the ocean velocity,**v**.

The power spectrum is calculated for the region denoted by the red box in Fig. 2. From the autocorrelation 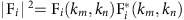 where *denotes the complex conjugate and *n*,*m* are gridpoints such that *n*=1,2,3…,*N m*=1,2,3…,*M* with *N*=*M* in this case. F_*i*_ is the Fourier transform of each component of the wind stress field denoted *i*=*x*,*y*.

The two-dimensional power spectrum is then calculated as 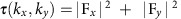 from which the one-dimensional power spectrum is computed by binning and averaging all components *N*_*k*_ with the same wavenumber 
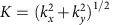



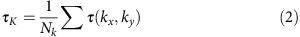


The one-dimensional power spectrum is calculated for each time step and then averaged over the total simulation time.

### Coherency magnitude squared

The magnitude-squared coherence was computed as 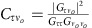, where 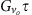 is the cross spectral density, and 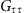
and 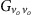 are the autospectrum of the wind stress and ocean surface velocity, respectively. Assuming a linear response between the wind and ocean velocities, 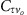
estimates the power transfer, where 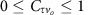, and a value of 1 indicates that the ocean velocity can be predicted entirely from the wind stress via a linear function.

### Code availability

The atmospheric component of the coupled model COSMO is available via http://www.cosmo-model.org with licenses depending on the intended application of the model. The ocean model (ROMS) is available, depending on the intended application of the model from the lead author D.B. on request. The coupler (OASIS) is available from https://verc.enes.org/oasis

### Data availability

The data that support the findings of this study are available from the corresponding author on request.

## Additional information

**How to cite this article:** Byrne, D. *et al.* Mesoscale atmosphere ocean coupling enhances the transfer of wind energy into the ocean. *Nat. Commun.* 7:11867 doi: 10.1038/ncomms11867 (2016).

## Supplementary Material

Supplementary Information Supplementary Figures 1-6.
