# Supplementary material for: Mesoscale atmosphere ocean coupling enhances the transfer of wind energy into the ocean
Source: Nat Commun. 2016 Jun 13;7:ncomms11867. doi: 10.1038/ncomms11867 (PMC4910005; doi:10.1038/ncomms11867)
Supplement: Supplementary Information — Supplementary Figures 1-6. [file ncomms11867-s1.pdf]

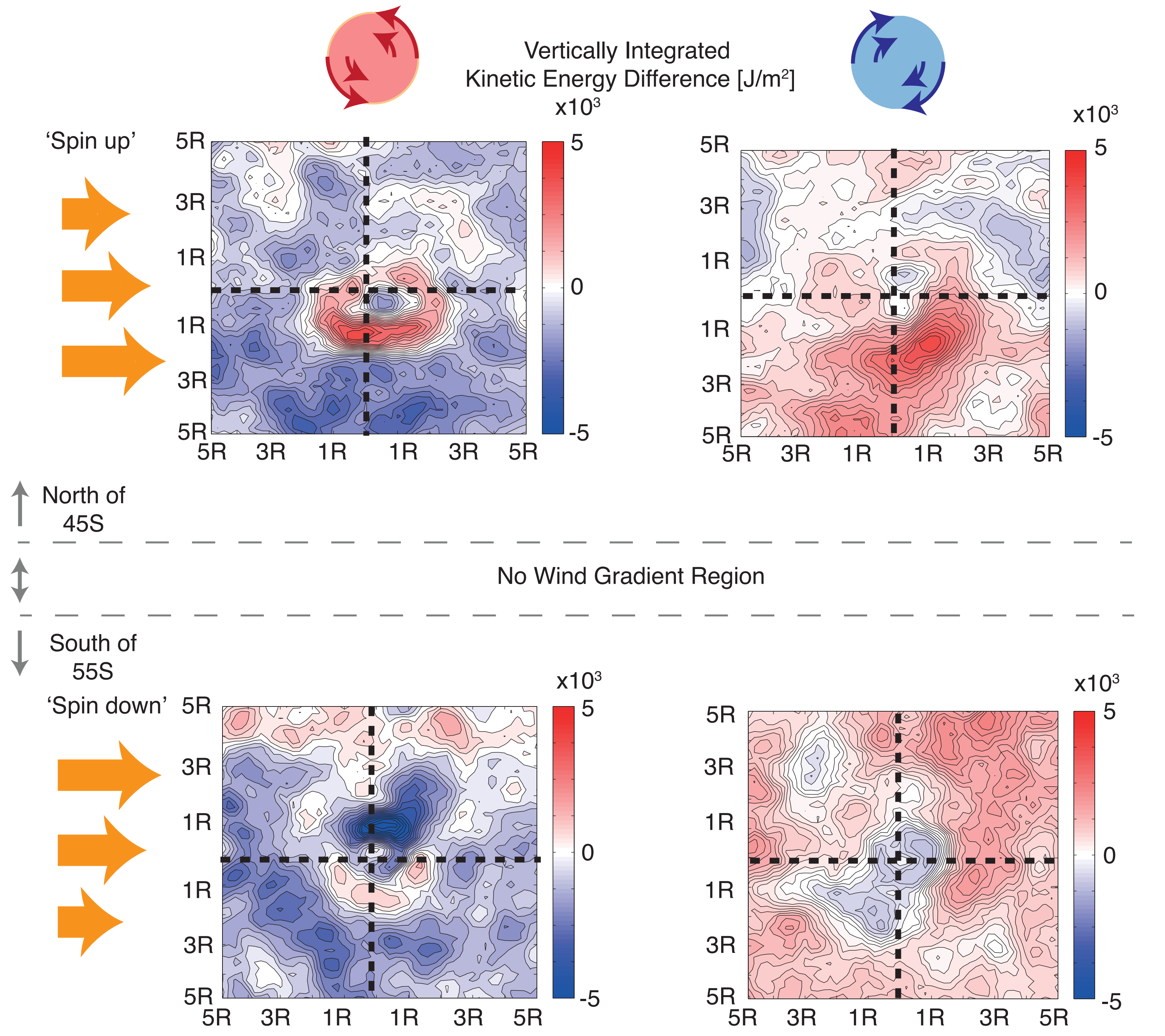

Supplementary Figure 1: Increased 'spin up' and 'spin down' signal is recovered if one only chooses eddies that reside in a wind gradient. Here chosen to avoid the peak in the cold core eddy abundance residing in no wind gradient.Regions North of 45S and South of 55S. (Mesoscale Fully Coupled - Mesoscale Mechanically Coupled)

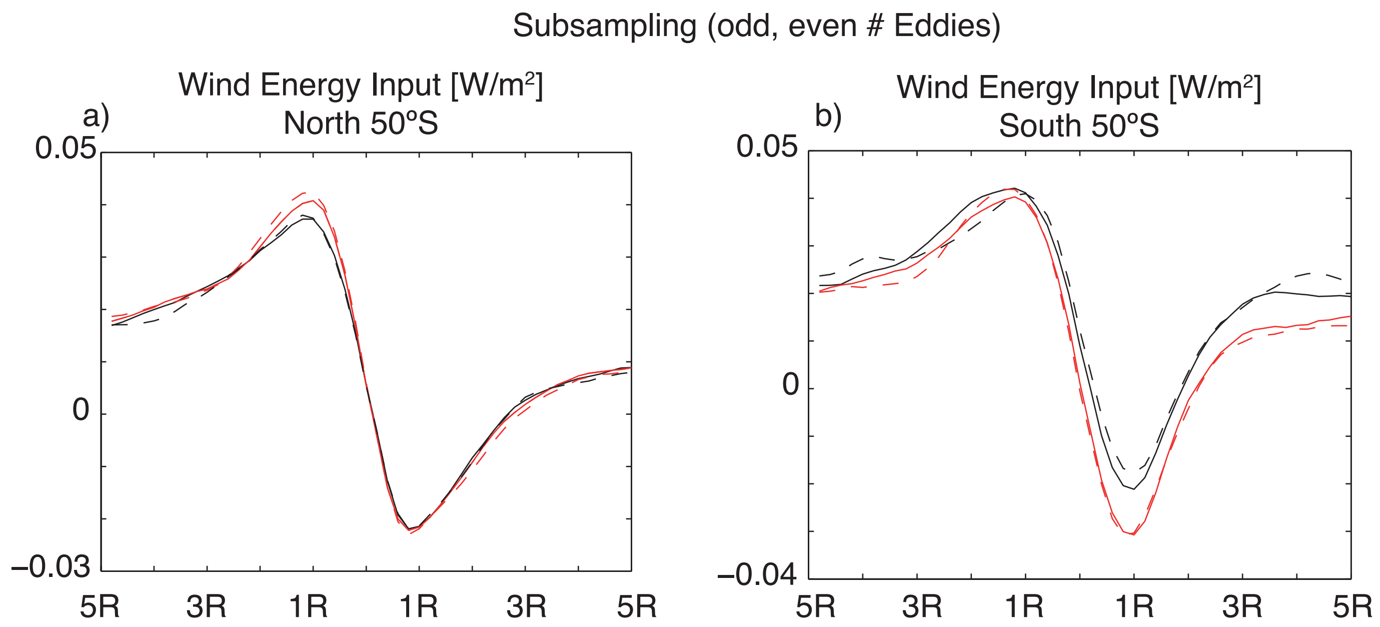

Supplementary Figure 2: Subsamples of eddy field for Fully Coupled (red) and Mechanically Coupled (black) with even number eddies (solid) andodd number eddies (dashed) for a) Northern spin up region, b) Southern spin down region.

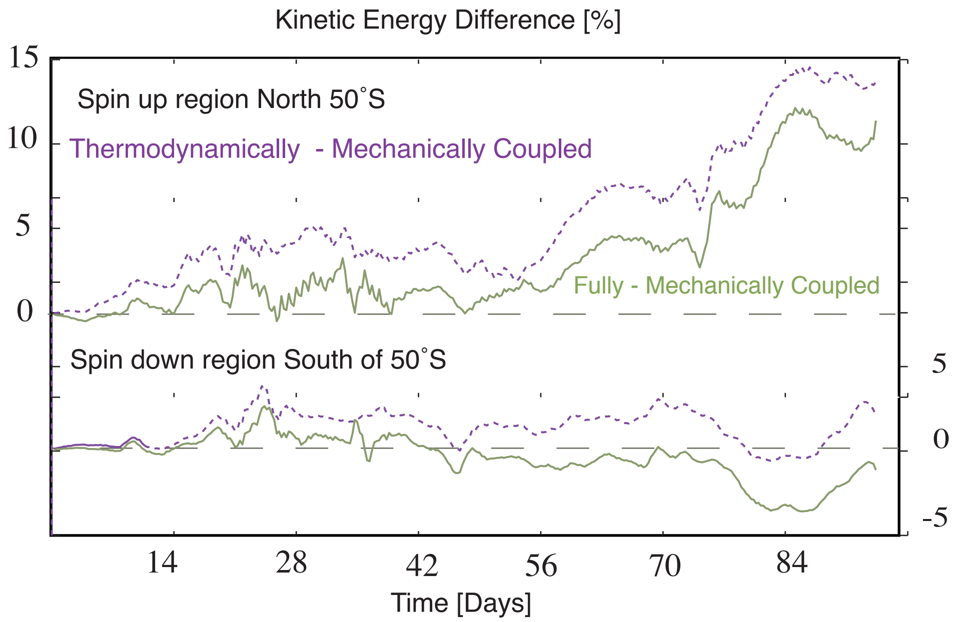

Supplementary Figure 3: Percentage change in volume integrated ocean kinetic energy for (>50°S) ‘spin up’ and (<50°S) ‘spin down’ region.
Mesoscale Thermodynamically Coupled - Mesoscae Mechanically Coupled simulations (dashed purple).
Fully Coupled - Mesoscale Mechanically Coupled (Solid Green)

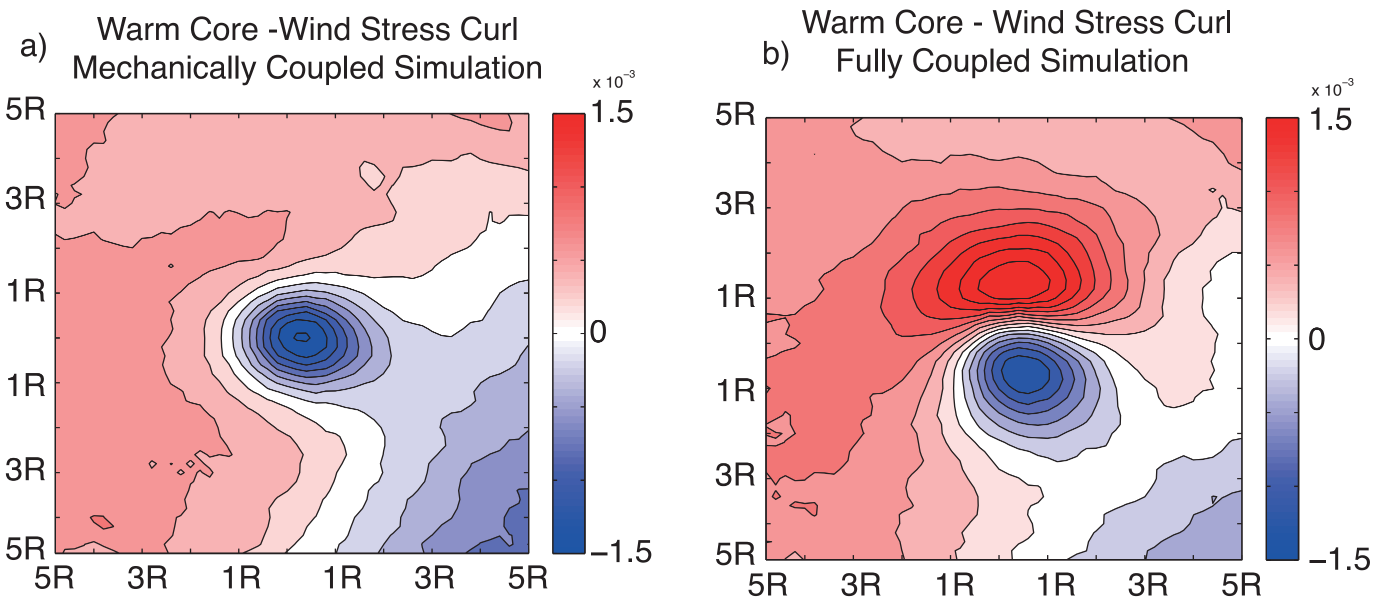

Supplementary Figure 4: Wind stress curl pattern for a) Mechannically Coupled b) Fully Coupled simulations. Dipole pattern of windstress curl indicitive that Thermodynamic effect is dominant.

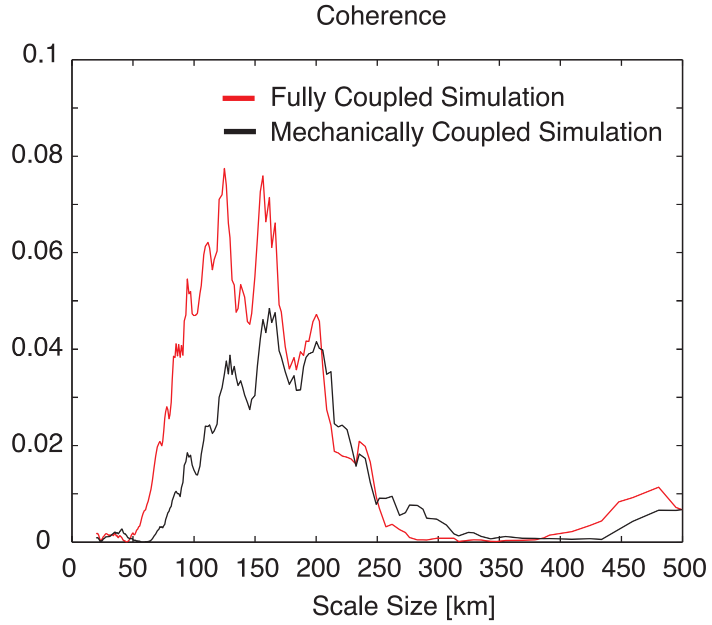

Supplementary Figure 5: Magnitude coherence spectrum for ocean surface velocity and wind stress. Mesoscale Fully Coupled (red) and Mesoscale Mechanically Coupled (black) indicates direct scale-scale wind forcing responsible for up to 8% of ocean kinetic energy at scales < 300km.

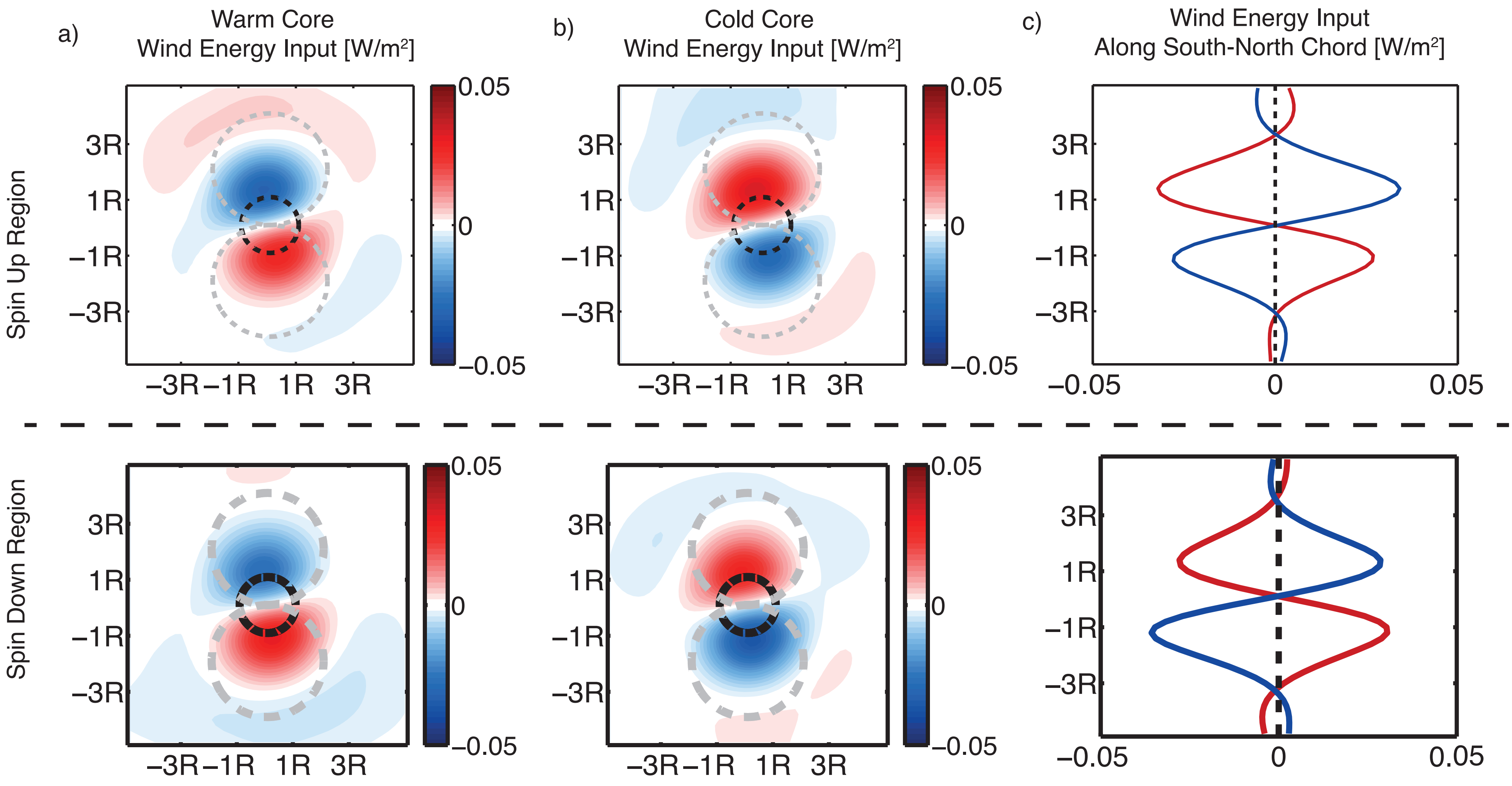

Supplementary Figure 6: Satellite Observation based analysis corresponding to Figure 3b&c. Eddy composites of wind energy input associated with geostrophic current anomalies for anticyclones (a) and cyclones (b) for eddies existing in meridional wind stress gradients greater than or less than 0.01 N m^{-2}. Eddies are normalized with their individual radii before averaging, R refers to eddy radius and only eddies with lifespans of at least two weeks are included in the analysis. (c) South-North Chords across the wind energy input eddy composites for anticyclones (red) and cyclones (blue).
